# Clinical and epidemiological profile of patients with accidental tetanus in a referral hospital: a 13-year historical series

**DOI:** 10.1007/s15010-026-02826-7

**Published:** 2026-05-18

**Authors:** Elaine Cristina de Oliveira Souza, Julia Guedes Valentim do Nascimento, Victor Ota, Arthur Duarte Fantesia Costa Cruz, Caroline Alves da Costa, Ana Luiza Martins de Oliveira, Claudia Caminha Escosteguy, Rafael Mello Galliez

**Affiliations:** 1 Serviço de Doenças Infecciosas e Parasitárias, Do Hospital Federal dos Servidores do Estado, Rua Sacadura Cabral, 178-Saúde, Rio de Janeiro, RJ 20221-161 Brazil; 2 Departamento de Doenças Infecciosas e Parasitárias, Da Faculdade de Medicina da Universidade Federal do Rio de Janeiro, Rua Professor Rodolpho Paulo Rocco, 13º andar, Campus da Universidade, Ilha do Fundão, Rio de Janeiro, RJ 21941-617 Brasil; 3 Escola Técnica do SUS, Da Secretaria Estadual de Saúde do Estado de Mato Grosso do Sul-ETSUS/SES/MS, Avenida Filinto Muller, 1480-Jardim Parati, Campo Grande, MS 79074-460 Brasil; 4 Fundação Carlos Chagas Filho de Amparo à Pesquisa, Do Estado do Rio de Janeiro-FAPERJ, Avenida Erasmo Braga, 118, 6º andar-Centro, Rio de Janeiro, RJ 20020-00 Brasil; 5 Instituto Estadual de Infectologia São Sebastião, Rua Sacadura Cabral, 178-Saúde, Rio de Janeiro, RJ 20221-161 Brasil; 6 Serviço de Epidemiologia, Do Hospital Federal dos Servidores do Estado, Rua Sacadura Cabral, 178-Saúde, Rio de Janeiro, RJ 20221-161 Brasil

**Keywords:** Tetanus, Intensive care units, Tracheostomy, Pneumonia, Ventilator-associated, Hospital mortality, Hospital-acquired infection

## Abstract

**Objective:**

To characterize the clinical profile, intensive care course, Ablett severity, healthcare-associated infections (HAIs), microbiology, and outcomes of patients with accidental tetanus treated at a Brazilian referral hospital over a 13-year period.

**Methods:**

We conducted a retrospective cohort study at the Instituto Estadual de Infectologia São Sebastião/Hospital Federal dos Servidores do Estado (Rio de Janeiro, Brazil). Consecutive patients aged > 28 days with accidental tetanus admitted between 2013 and 2025 were included according to national surveillance criteria. Categorical variables were summarized as counts and percentages, and continuous variables as means and standard deviations or medians and interquartile ranges (IQR), as appropriate. Associations with in-hospital mortality were explored in univariate analyses using the Fisher exact test and crude odds ratios (OR) with 95% confidence intervals (95% CI).

**Results:**

Thirty patients were included; most were male and urban residents, with a median age of 43 years (IQR 29.0–60.8). ICU admission occurred in 28/30 (93.3%); 25/30 (83.3%) required mechanical ventilation and 24/30 (80%) underwent tracheostomy. ICU length of stay was prolonged (median 28.0 days, IQR 13.0–44.8). HAIs occurred in 21/30 (70%), predominantly ventilator-associated pneumonia (20/30; 66.7%); the most frequent pathogens were *Acinetobacter baumannii*, *Pseudomonas aeruginosa*, and *Staphylococcus aureus*. In-hospital mortality was 8/30 (26.7%), concentrated among patients aged ≥ 60 years (OR 6.25, 95% CI 1.02–38.08) and among those with Ablett grade IV disease (8/18; 44.4%).

**Conclusion:**

Severe accidental tetanus was associated with prolonged ICU stay, high ventilatory support requirements, and a substantial VAP-related infection burden, supporting catch-up vaccination strategies and rigorous airway management, ventilator-weaning, VAP-prevention, and antimicrobial-stewardship practices.

## Background

Tetanus is a vaccine-preventable acute infection caused by *Clostridium tetani*, which persists as a serious public-health problem in many low- and middle-income countries despite decades of immunization efforts [1–3]. While maternal and neonatal tetanus have markedly declined, accidental tetanus still drives substantial demand for intensive care, with prolonged lengths of stay (LOS) and high resource use [1–4].

In high-income countries, vaccine-preventable bacterial diseases such as tetanus and diphtheria are rare, although pertussis persists in some settings, such as the United States and the United Kingdom [5]. In contrast, in many low- and middle-income countries, tetanus is still endemic [5], such as in India [6], Jamaica [7], and Ethiopia [8]. In Brazil, national surveillance shows declining incidence but sustained morbidity and mortality, underscoring the importance of ICU-focused descriptions from South American countries [4–9].

The clinical course is driven by tetanospasmin-mediated neurotoxicity, shorter incubation and progression times are linked to more severe disease and worse outcomes [2]. Critical-care management commonly requires early airway strategies (including tracheostomy) and prolonged mechanical ventilation, with risks of Healthcare-Associated Infection (HAIs) [1, 10]. International series reveal wide heterogeneity across settings, in age/sex profiles, ventilatory support, HAI burden, LOS and mortality, spanning high-income (e.g., France, Japan) [11, 12] and middle-income contexts (e.g., Slovenia, Romania and African hospitals) [13–15]. These differences suggest an important role of care processes, beyond patient mix, in shaping outcomes.

In Brazil, the high severity, case fatality, morbidity, and resource intensity of tetanus care make it a public-health priority; the condition is legally notifiable, with mandatory reporting by public and private services to the Brazilian National Notifiable Diseases Information System (*Sistema de Informação de Agravos de Notificação*) to guide planning, interventions, and impact evaluation across municipalities and states [16].

Accidental tetanus, despite a significant reduction over the years, remains a public health problem in Brazil, mainly due to the severity of the disease, its complications, and high costs [4]. Few studies have described tetanus in the ICU in detail, quantifying ventilatory support, tracheostomy, HAI burden, length of stay, and mortality. In this context, characterizing tetanus managed in Brazilian ICUs may inform both prevention strategies (vaccination and wound care) and in-hospital care processes.

The aim of this study was to characterize the clinical profile, intensive care course, healthcare-associated infections, microbiology, and in-hospital outcomes of patients with accidental tetanus treated at a Brazilian referral hospital over a 13-year period.

## Methods

### Study design and setting

This was a single-hospital retrospective cohort study of consecutive patients admitted with suspected or confirmed accidental tetanus between January 1, 2013, and August 31, 2025. The study took place at the Instituto Estadual de Infectologia São Sebastião (IEISS), currently operating within the Hospital Federal dos Servidores do Estado, a tertiary-level general hospital and state referral center for infectious diseases in Rio de Janeiro, Brazil. Patients are referred to IEISS through the Rio de Janeiro State Regulation System based on clinical judgement regarding the need for isolation and/or intensive care.

### Participants and case definition

Eligible participants were consecutive patients older than 28 days, admitted between January 1, 2013, and August 31, 2025, with suspected or confirmed accidental tetanus, who met the Brazilian National Notifiable Diseases Information System clinical case definition. According to this definition, eligible cases included individuals who presented one or more compatible clinical features, such as trismus, dysphagia, sardonic grin, opisthotonus, or localized or generalized muscle rigidity/spasms, regardless of vaccination history, previous tetanus episodes, or identification of an entry wound. Neonatal tetanus and non-accidental forms of tetanus were not eligible. Follow-up extended from hospital admission to hospital discharge or death.

### Data sources and variables

Data were extracted from multidisciplinary clinical records and tetanus notification forms by trained investigators using a standardized form. The variables collected were grouped as follows:

1. Sociodemographic characteristics: biological sex; race/skin color; employment status; marital status; age group, place of birth (state), residence, sanitation, and vaccination record;
2. Injury characteristics: portal of entry and anatomical site of inoculation;
3. Clinical course and severity: symptom onset, time from injury to symptom onset, time from injury to treatment start, time from symptom onset to treatment start, and Ablett grade;
4. Tetanus-directed treatment: wound care, tetanus immunotherapy and antibiotic therapy;
5. Healthcare-associated infections and microbiology: occurrence and type of healthcare-associated infection, microbiological isolates, and antimicrobial resistance profile when available; and.
6. Outcomes: ICU admission, ICU length of stay, hospital length of stay, sequelae at discharge, and in-hospital mortality.

Tetanus severity was classified according to the Ablett grading system (grades I–IV), in which higher grades indicate greater clinical severity [8]. Ablett grade was obtained from the medical record when explicitly documented or assigned retrospectively from the recorded clinical description when sufficient information was available.

Autonomic dysfunction was operationally inferred from the use of magnesium sulfate during hospitalization after chart review. At our institution, magnesium sulfate is part of the standard therapeutic protocol for patients with clinical manifestations compatible with autonomic dysfunction, including hemodynamic instability, persistent tachycardia, labile hypertension, and profuse sweating. Therefore, in this analysis, magnesium sulfate use was adopted as an indirect marker of autonomic dysfunction and should be interpreted with caution.

For comparative analyses, selected continuous variables were categorized a priori (e.g., time intervals and MV days by sample median; age as < 18, 18–59, ≥ 60 years; race as White vs. non-White; employment as yes vs. no; marital status as married/partnered vs. other). The full categorization schema is provided in the appendix (Table 1).

### Outcomes

The prespecified outcomes were ICU length of stay and in-hospital mortality. Secondary outcomes included hospital length of stay, need for mechanical ventilation, duration of mechanical ventilation, tracheostomy, and healthcare-associated infections. ICU length of stay was calculated in complete 24-hour blocks; stays shorter than 24 h were recorded as 0 days.

### Statistical analysis

Categorical variables were summarized as counts and percentages, and continuous variables as means and standard deviations or medians and interquartile ranges (IQR), as appropriate. Associations between selected variables and in-hospital mortality were explored in univariate analyses using the Fisher exact test and crude odds ratios (OR) with 95% confidence intervals (95% CI). Because of the small sample size and the limited number of outcome events, no multivariable model was fitted. Statistical significance was assessed at a two-sided alpha level of 0.05. Analyses were performed in R (R Core Team, 2024) [17].

## Results

Thirty patients were included; most were male (24/30, 80%) and urban residents (23/30, 76.7%). Median age was 43 years (IQR 29.0–60.8). Puncture injury was the most common portal of entry (25/30, 83.3%), typically in the lower limbs (foot and leg). Vaccination history was incomplete; most patients (80%) had either no documented prior vaccination or no vaccination record available. Exploratory univariate associations with in-hospital mortality, expressed as crude odds ratios (OR) with 95% confidence intervals (95% CI), are shown in Table 1.

**Table 1:** Exploratory univariate analysis of factors associated with in-hospital mortality among patients with accidental tetanus treated at a Brazilian referral hospital, Rio de Janeiro, Brazil, 2013–2025 (*n* = 30)

Variable	Category	Total *n* (%)	Outcome		OR (95% CI)
			Discharged *n* (%)	Death *n* (%)	
*Sociodemographic characteristics*					
Patients		30 (100%)	22 (73.3%)	8 (26.7%)	–
Biological sex	Male	24 (80%)	19 (79.2%)	5 (20.8%)	1.00
	Female	6 (20%)	3 (50%)	3 (50%)	3.80 (0.58–24.88)
Race/skin color	Non-white	16 (53.3%)	11 (68.8%)	5 (31.2%)	1.52 (0.28–8.03)
	White	13 (43.3%)	10 (76.9%)	3 (23.1%)	1.00
	Missing	1 (3.3%)	1 (100%)	0 (0%)	–
Employment status	Yes	20 (66.7%)	17 (85.0%)	3 (15%)	1.00
	No	10 (33.3%)	5 (50%)	5 (50%)	5.67 (0.99–32.43)
Marital status	Separated/single/widowed	18 (60%)	13 (72.2%)	5 (27.8%)	3.08 (0.30–31.33)
	Married/partnered	9 (30%)	8 (88.9%)	1 (11.1%)	1.00
	Missing	3 (10%)	1 (33.3%)	2 (66.7%)	–
Age group	< 18 years	3 (10%)	3 (100%)	0 (0%)	–
	18–59 years	18 (60%)	15 (83.3%)	3 (16.7%)	1.00
	≥ 60 years	9 (30%)	4 (44.4%)	5 (55.6%)	6.25 (1.02–38.08)
Place of birth	Rio de Janeiro (state)	18 (60%)	11 (61.1%)	7 (38.9%)	7.00 (0.73–66.80)
	Other Brazilian states	12 (40%)	11 (91.7%)	1 (8.3%)	1.00
Residence	Informal settlement	3 (10%)	3 (100%)	0 (0%)	–
	Urban	23 (76.7%)	16 (69.6%)	7 (30.4%)	1.00
	Rural	2 (6.7%)	1 (50%)	1 (50%)	2.28 (0.12–41.99)
	Missing	2 (6.7%)	2 (100%)	0 (0%)	
Basic sanitation	Yes	26 (86.7%)	18 (69.0%)	8 (30.8%)	
	Missing	3 (10%)	3 (100%)	0 (0%)	–
	No	1 (3.3%)	1 (100%)	0 (0%)	–
Vaccination record	Not vaccinated	15 (50%)	12 (80%)	3 (20%)	1.00 (0.08–12.56)
	1 documented dose	5 (16.7%)	4 (80%)	1 (20%)	1.00
	3 documented doses	1 (3.3%)	1 (100%)	0 (0%)	–
	Missing	9 (30%)	5 (55.6%)	4 (44.4%)	–
*Injury characteristics*					
Portal of entry—puncture		25 (83.3%)	17 (68.0%)	8 (32%)	
Portal of entry—abrasion		5 (16.7%)	3 (60%)	2 (40%)	2.11 (0.28–15.77)
Portal of entry—laceration		3 (10%)	1 (33.3%)	2 (66.7%)	7.00 (0.54–91.12)
Portal of entry—burn		2 (6.7%)	2 (100%)	0 (0%)	
Portal of entry—surgical		1 (3.3%)	0 (0.0%)	1 (100%)	
Anatomical site—lower limbs		23 (79.3%)	17 (73.9%)	6 (26.1%)	0.71 (0.11–6.03)
Anatomical site—upper limbs		8 (27.6%)	4 (50.0%)	4 (50.0%)	4.25 (0.73–26.93)
Anatomical site—head		1 (3.4%)	1 (100%)	0 (0.0%)	–
Anatomical site—others		1 (3.4%)	1 (100.0%)	0 (0.0%)	–
Clinical course and severity*					
Symptom—trismus		25 (83.3%)	18 (72%)	7 (28%)	1.56 (0.19–33.30)
Symptom—dysautonomy		24 (80%)	18 (75.0%)	6 (25.0%)	0.67 (0.10–5.68)
Symptom—neck stiffness		17 (56.7%)	12 (70.6%)	5 (29.4%)	1.39 (0.27–8.16)
Symptom—limb rigidity		14 (46.7%)	12 (85.7%)	2 (14.3%)	0.28 (0.04–1.52)
Symptom—abdominal rigidity		12 (40%)	10 (83.3%)	2 (16.7%)	0.40 (0.05–2.20)
Symptom—opisthotonus		11 (36.7%)	7 (63.6%)	4 (36.4%)	2.14 (0.40–11.74)
Symptom—spasms		11 (36.7%)	9 (81.8%)	2 (18.2%)	0.48 (0.06–2.67)
Symptom—generalized muscle rigidity		8 (26.7%)	5 (62.5%)	3 (37.5%)	2.04 (0.33–11.84)
Symptom—risus sardonicus		4 (13.3%)	3 (75.0%)	1 (25%)	0.90 (0.04–8.53)
Symptom—fever		5 (16.7%)	4 (80%)	1 (20%)	0.64 (0.03–5.38)
Symptom—other		26 (86.7%)	18 (69.2%)	8 (30.8%)	
Ablett classification	II	5 (16.7%)	5 (100%)	0 (0%)	–
	III	7 (23.3%)	7 (100%)	0 (0%)	
	IV	18 (60%)	10 (55.6%)	8 (44.4%)	
Time from injury to treatment start	≤ 10 days (median)	13 (43.3%)	9 (69.2%)	4 (30.8%)	4.89 (0.46–51.87)
	> 10 days	12 (40%)	11 (91.7%)	1 (8.3%)	1.00
	Missing	5 (16.7%)	2 (40%)	3 (60%)	–
Time from symptom onset to treatment start	≤ 3 days (median)	20 (66.7%)	16 (80%)	4 (20%)	1.00
	> 3 days	10 (33.3%)	6 (60%)	4 (40%)	2.67 (0.50–14.22)
Time from injury to symptom onset	≤ 6 days (median)	18 (60%)	13 (72.2%)	5 (27.8%)	–
	> 6 days	7 (23.3%)	7 (100%)	0 (0%)	
	Missing	5 (16.7%)	2 (40%)	3 (60%)	–
*Infections*					
Healthcare-associated infection		21 (70%)	16 (76.2%)	5 (23.8%)	0.63 (0.11–3.80)
Ventilator-associated pneumonia		20 (66.7%)	15 (75%)	5 (25%)	0.78 (0.14–4.66)
Bloodstream infection		5 (16.7%)	4 (80%)	1 (20%)	0.64 (0.03–5.38)
Urinary tract infection		6 (20%)	5 (83.3)	1 (16.7)	0.48 (0.05–4.94)
*Tetanus-directed treatment and organ support*					
Number of debridements	0	1 (3.3%)	1 (100%)	0 (0%)	–
	1	17 (56.7%)	12 (70.6%)	5 (29.4%)	1.00
	2	10 (33.3%)	7 (70%)	3 (30%)	1.02 (0.19–5.68)
	Missing	2 (6.7%)	2 (100%)	0 (0%)	–
Antibiotic use during admission		25 (83.3%)	18 (72%)	7 (28%)	1.56 (0.19–33.30)
Number of distinct antibiotics used	≤ 1.5 (median)	15 (50%)	12 (80%)	3 (20%)	1.00
	> 1.5	15 (50%)	10 (66.7%)	5 (33.3%)	2.00 (0.38–10.51)
Penicillin use for *C. tetani* treatment		14 (46.7%)	11 (78.6%)	3 (21.4%)	0.60 (0.10–3.07)
Human anti-tetanus immunoglobulin		29 (96.7%)	21 (72.4%)	8 (27.6%)	–
Metronidazole use for *C. tetani* treatment		25 (83.3%)	18 (72%)	7 (28%)	1.56 (0.19–33.30)
Tetanus-diphtheria vaccine		25 (83.3%)	20 (80%)	5 (20%)	0.17 (0.02–1.25)
Tetanus antitoxin		18 (60%)	13 (72.2%)	5 (27.8%)	1.15 (0.22–6.80)
Tetanus toxoid administered during admission		3 (10%)	3 (100%)	0 (0%)	–
*ICU course and organ support*					
ICU admission		28 (93.3%)	21 (75%)	7 (25%)	0.33 (0.01–9.17)
Mechanical ventilation		25 (83.3%)	17 (68%)	8 (32%)	
Tracheostomy performed		24 (80%)	17 (70.8%)	7 (29.2%)	2.06 (0.26–43.23)
ICU length of stay (days)	≤ 28 days (median)	14 (50%)	12 (85.7%)	2 (14.3%)	
	> 28 days	14 (50%)	9 (64.3%)	5 (35.7%)	3.33 (0.57–27.24)
Hemodialysis during admission		9 (30%)	5 (55.6%)	4 (44.4%)	3.40 (0.61–20.03)

Data are presented as n (%) unless otherwise indicated. Continuous variables are presented as median (IQR). Odds ratios are crude estimates from univariate analyses. ORs were not calculated when one category contained all events (100%) or no events (0%). ICU length of stay was calculated in complete 24-hour blocks; stays shorter than 24 h were recorded as 0 days. Healthcare-associated infections were those recorded by the hospital infection-control team during admission. Race/skin color follows the Brazilian census categories

*OR* odds ratio, *CI* confidence interval

*A single patient may present more than one response category. Vaccination history was incomplete; most patients had either no documented prior vaccination or no vaccination record available. Autonomic dysfunction was operationally inferred from magnesium sulfate use during hospitalization after chart review

Cardinal signs at presentation included trismus (25/30; 83.3%), neck stiffness (17/30; 56.7%), and opisthotonus (11/30; 36.7%). Most patients were admitted to the ICU (28/30; 93.3%); 25/30 (83.3%) required MV and 24/25 (96%) underwent tracheostomy among those with available data (complete-case). HAIs occurred in 21/30 (70%). The injury-to-treatment and symptom-to-treatment intervals were 10 days (IQR 7–14) and 3 days (IQR 1–4.8), respectively. Patients received a median of 1.5 distinct antibiotics during admission (IQR 1–2); 25/30 (83.3%) received at least one antibiotic, and 14/30 (46.7%) received penicillin. ICU LOS was 28 days (IQR 13–44.8). Ventilator-associated pneumonia occurred in 20/30 (66.7%), with bloodstream infection reported in 5/30 (16.7%) and urinary tract infection in 6/30 (20%) (Table 1).

In-hospital mortality was 8/30 (26.7%). Deaths were concentrated among older patients (≥ 60 years: OR 6.25, 95% CI 1.02–38.08) and among those with Ablett grade IV disease. Among patients with Ablett grade IV, 8/18 (44.4%) died, whereas no deaths occurred among those with Ablett grades II–III (Table 2). All patients who died required mechanical ventilation and tracheostomy. Only one patient (1/8, 12.5%) did not undergo tracheostomy because she died before the procedure.

**Table 2:** Characteristics of non-survivors among patients with accidental tetanus treated at a Brazilian referral hospital, Rio de Janeiro, Brazil, 2013–2025 (*n* = 8)

Variable	Patients							
	1	2†	3	4	5	6	7	8
Biological sex	Male	Female	Male	Female	Male	Male	Male	Female
Race/skin color	White	Not white	Not white	White	White	Not white	Not white	Not white
Economically active	No	No	Yes	No	Yes	Yes	Yes	No
Age	72	58	62	74	38	21	82	79
Residence	Urban	Urban	Rural	Urban	Urban	Urban	Urban	Urban
Basic sanitation	Yes	Yes	Yes	Yes	Yes	Yes	Yes	Yes
Incubation period	5	7	Missing	7	Missing	6	4	Missing
Vaccination record	1 documented dose	No record available/missing	No record available/missing	Not vaccinated	No record available/missing	Not vaccinated	Not vaccinated	No record available/missing
Time from symptom onset to treatment (days)	8	7	Missing	11	Missing	6	8	Missing
Time from injury to treatment (days)	3	0	34	4	1	0	4	15
Hemodialysis during admission	Yes	No	No	No	Yes	No	Yes	Yes
Antibiotic use	Yes	No	Yes	Yes	Yes	Yes	Yes	Yes
Penicillin use	No	No	No	Yes	No	Yes	Yes	No
ICU admission	Yes	No	Yes	Yes	Yes	Yes	Yes	Yes
ICU length of stay (days)	42	0	6	4	83	72	53	32
Mechanical ventilation	Yes	Yes	Yes	Yes	Yes	Yes	Yes	Yes
Tracheostomy performed	Yes	No	Yes	Yes	Yes	Yes	Yes	Yes
Ablett grade	4	4	4	4	4	4	4	4

“Missing” indicates data not recorded in the chart. “Brown (Pardo)” follows the Brazilian census category

^†^One non-surviving patient (female, 58 years) was not classified as admitted to the ICU because she died before completing 24 h in the unit. She received mechanical ventilation while awaiting ICU transfer; therefore, mechanical ventilation was recorded as “Yes,” ICU admission as “No,” and ICU length of stay as 0 days

Healthcare-associated infections occurred in 21/30 (70%). Single-organism cultures were identified in 6/21 patients, whereas polymicrobial profiles were identified in 15/21. The most frequently identified organisms were *A. baumannii* (37%), *S. aureus* (33%) and *P. aeruginosa* (27%) (Table 3).

**Table 3:** Descriptive microbiological profile and infection characteristics among patients with accidental tetanus treated at a Brazilian referral hospital, Rio de Janeiro, Brazil, 2013–2025 (*n* = 30)

Variable	Total *n* (%)	Outcome		OR (95% CI)
		Discharged *n* (%)	Death *n* (%)	
*Infection*				
No	9 (30%)	6 (67%)	3 (33%)	1.00
Yes	21 (70%)	16 (76.2%)	5 (23.8%)	0.63 (0.11–3.80)
*Number of microbial agents*				
None	9 (30%)	6 (67%)	3 (33%)	1.00
Monomicrobial infection	6 (20%)	6 (100%)	0 (0%)	
Polymicrobial infection	15 (50%)	10 (67%)	5 (33%)	–
Bacterial agents*				
*A. baumannii*	11 (37%)	8 (72.73%)	3 (27.27%)	1.05 (0.18–5.53)
*P. aeruginosa*	8 (27%)	6 (75.0%)	2 (25.0%)	0.89 (0.11–5.26)
*S. aureus*	10 (33%)	9 (90%)	1 (10%)	0.21 (0.01–1.46)
*K. pneumoniae*	3 (10%)	1 (33%)	2 (67%)	7.00 (0.58–167.01)
*P. mirabilis*	2 (7%)	1 (50%)	1 (50%)	3.00 (0.11–83.00)
Other MDRO	13 (43%)	8 (62%)	5 (38%)	–
Infection caused by carbapenem-resistant bacteria*				
Carbapenem-resistant *K. pneumoniae*	3 (10%)	1 (33%)	2 (67%)	7.00 (0.58–167.01)
Carbapenem-resistant *A. baumannii*	11 (36.6%)	7 (64%)	4 (36%)	2.14 (0.40–11.74)
Carbapenem-resistant *P. aeruginosa*	2 (6.6%)	1 (50%)	1 (50%)	3.00 (0.11–83.00)
Carbapenem-resistant *P. mirabilis*	1 (3.3%)	0 (0%)	1 (100%)	–

Data are presented as n (%). Patients could have more than one microbiological isolate and more than one type of infection. Percentages are based on the total cohort unless otherwise specified

*MDRO* Multidrug-Resistant Organisms

*A single patient may present more than one response category

## Discussion

In this 13-year single-hospital cohort, we characterized the clinical profile, intensive care course, healthcare-associated infections, microbiology, and outcomes of patients with accidental tetanus treated at a Brazilian referral hospital. The cohort showed a predominantly male profile and a median age of 43 years (IQR 29.0–60.8), with high use of organ support and a substantial burden of healthcare-associated infections. Mechanical ventilation was required in 25/30 patients (83.3%) and tracheostomy in 24/30 (80%); median ICU length of stay was 28 days, and in-hospital mortality reached 8/30 (26.7%). Human anti-tetanus immunoglobulin was administered in most cases, whereas tetanus toxoid administered during admission was less frequent, reflecting the distinction between passive immunization and in-hospital vaccine updating. Taken together, these findings depict a resource-intensive care pathway with prolonged ICU stay and clinically significant fatality, consistent with the recognized severity of tetanus in referral settings.

Severity was concentrated in the highest Ablett grade and aligned with organ-support intensity: all deaths occurred among patients with Ablett grade IV disease, and invasive ventilation plus tracheostomy were frequent, helping to explain the prolonged ICU course and mortality profile observed at the study site. These patterns reinforce the need for early airway strategies and structured weaning in severe tetanus.

Across Brazil, the disease presents a stable clinical-epidemiological profile: marked male predominance, concentration in middle-aged and older adults, higher occurrence among brown/black individuals, with urban residence, occupational exposures linked to construction and agriculture, penetrating wounds, with the entry point typically in the lower limbs and incomplete or absent vaccination [18, 19].

The health crisis of the COVID-19 pandemic imposed multifactorial barriers in the country, including social isolation, the overload of basic health units, and the reallocation of health professionals to combat the disease in emergency services, which resulted in underreporting and a drastic reduction in spontaneous demand for routine immunization between 2019 and 2022. Furthermore, the rise of vaccine hesitancy movements (anti-vaccine campaigns) and the systematic dissemination of misinformation (fake news) about the safety of immunizations aggravated the scenario of public distrust and compromised vaccination coverage [20].

In Brazil, hospital LOS for tetanus averaged 16.79 days between 2008 and 2019, rising to 18.67 days in the Southeast and reaching 30.11 days in Rio de Janeiro, a pattern consistent with the prolonged ICU stay and high resource use observed in the present study [21]. Concomitantly, vaccination coverage for tetanus-containing vaccines fell short of targets during 2019–2021, with Rio de Janeiro consistently below national averages, reinforcing system-level contributors to delayed care and extended admissions [4].

The infection scenario was dominated by ventilator-associated pneumonia and non-fermenting Gram-negative bacilli (*A. baumannii*,* P. aeruginosa*), underscoring the centrality of VAP prevention bundles and antimicrobial stewardship to shorten ICU trajectories in severe tetanus. Recent studies corroborate this ecology and recommend bundle-based prevention coupled with short-course, de-escalated antibiotic regimens when appropriate, to minimize exposure without compromising outcomes and to shorten ICU trajectories in severe tetanus [22, 23].

International comparisons reveal marked heterogeneity across settings. A French multicenter ICU series (2000–2014) reported near-universal MV (90%), long ventilation duration (median 36 days), frequent HAIs (61%), and low ICU mortality (14%) [11]. Nationwide analyses from Japan (2010–2016) described an older case-mix, lower MV use overall, high tracheostomy among ventilated patients, prolonged hospital LOS, and low in-hospital mortality [12]. In Slovenia, a national cohort reported very high rates of MV and HAIs with long ICU LOS and intermediate mortality [13]. Romanian surveillance (2010–2023) showed high case-fatality in predominantly rural males and children [14]. A meta-analysis from African hospitals estimated pooled adult tetanus mortality above 40% amid limited access to ICU and ventilation [15]. Compared with these reports, the present cohort showed a younger, predominantly male profile with high ventilatory support and substantial HAI burden, aligning with prolonged ICU LOS and an intermediate fatality, and suggesting that, in addition to patient characteristics, care processes, airway strategies, infection prevention, and ICU organization, shape outcomes.

Determinants of prolonged ICU stay appear to be multifactorial. Prospective work from Vietnam linked initial severity (older age, deranged vital signs, hypoxaemia, shorter symptom-to-admission interval) to MV and autonomic dysfunction, with downstream functional limitations at six months, highlighting the impact of severe physiology and complications on resource use and recovery [24]. In an emergency-ICU cohort from China, MV and tracheostomy were independently associated with longer intensive-care duration (lower hazard of discharge), which is consistent with the present findings, though these interventions are also proxies of severity, introducing treatment-by-indication confounding [25]. Critical-care literature further suggests that protocolized care (timely tracheostomy in selected patients, structured weaning, and strict VAP-prevention bundles can shorten ventilation and ICU stay in appropriate contexts, an actionable hypothesis for middle-income referral ICUs.

Beyond initial severity, process-of-care factors are actionable targets. Reviews emphasize early elective tracheostomy to reduce aspiration risk, high-dose benzodiazepines as the backbone of spasm control with neuromuscular blockade and mechanical ventilation for refractory spasms, and rigorous prevention of ventilator-associated events [26]. In settings similar to ours, standardizing criteria and timing for tracheostomy, structuring ventilator-weaning protocols, and implementing prevention bundles are plausible strategies to shorten ICU stay without compromising safety.

Finally, men’s underuse of health services, shaped by masculinities that delay help-seeking and weaken primary-care linkage, helps explain male under-vaccination; targeted engagement of men in preventive care (extended hours, workplace outreach, and routine immunization checks) should be integrated into tetanus control strategies [27–29]. Aligning these actions with catch-up vaccination could reduce preventable ICU demand in Brazil [4].

At the bedside, priorities include (i) standardized airway strategies (clear criteria and timing for tracheostomy), (ii) objective sedation/neuromuscular blockade targets with early mobilization where feasible, (iii) structured ventilator-weaning protocols, and (iv) consistent implementation of HAI-prevention bundles. At the system level, strengthening immunization, with targeted male engagement and routine immunization checks across primary, emergency, and occupational health services, remains essential to reduce preventable ICU demand. Implementing a prospective micro-observatory for length of stay, ventilator days, and HAI density would strengthen information quality, operationalize bedside decision-making, and provide a benchmarked evidence base for policy formulation and programmatic action at national and international levels.

Limitations include the single-center design, small sample size, and retrospective data, which limit statistical power and generalizability; missing vaccination histories and potential information bias are acknowledged. In addition, autonomic dysfunction was defined retrospectively through a treatment-based proxy (magnesium sulfate use), and some degree of misclassification cannot be excluded. Nonetheless, congruence with external evidence supports the credibility of the conclusions and identifies concrete targets for practice improvement and policy action.

## Conclusions

Accidental tetanus in this Brazilian referral hospital was highly resource-intensive, with frequent ventilatory support (mechanical ventilation, 83.3%; tracheostomy, 80%), a substantial burden of healthcare-associated infections (70%), prolonged ICU stay (median 28 days; mean 31.7), and in-hospital mortality of 26.7%. Variation across settings suggests that outcomes reflect not only patient characteristics but also care processes. Taken together with the concentration of deaths among patients with Ablett grade IV disease and the predominance of ventilator-associated infections, these findings support prioritizing early airway protocols, structured ventilator weaning, and rigorous VAP prevention to safely reduce ICU length of stay. At the system level, strengthening immunization, with targeted engagement of men and routine vaccination checks at points of care, remains essential to reduce the preventable burden of tetanus and ICU demand in the Americas.

## Data Availability

No datasets were generated or analysed during the current study.

## Declarations

### Declarations of generative AI and AI-assisted technologies in the manuscript preparation process

During the preparation of this work, the authors used ChatGPT Plus, version 5.2-Thinking (OpenAI San Francisco, CA, USA) for language editing (grammar, clarity, and adherence to formal academic register) and for translation from Brazilian Portuguese into English. After using this tool/service, the authors reviewed and edited the content as needed and take full responsibility for the final manuscript.
